# Breast vibro-acoustography: initial results show promise

**DOI:** 10.1186/bcr3323

**Published:** 2012-09-29

**Authors:** Azra Alizad, Dana H Whaley, Matthew W Urban, Rickey E Carter, Randall R Kinnick, James F Greenleaf, Mostafa Fatemi

**Affiliations:** 1 Department of Physiology and Biomedical Engineering, Mayo Clinic, 200 First Street SW, Rochester, MN 55905, USA; 2 Department of Internal Medicine, Mayo Clinic, 200 First Street SW, Rochester, MN 55905, USA; 3 Department of Radiology, Mayo Clinic, 200 First Street SW, Rochester, MN 55905, USA; 4 Division of Biomedical Statistics and Informatics, Mayo Clinic, 200 First Street SW, Rochester, MN 55905, USA

**Keywords:** acoustic imaging, breast lesions, radiation force breast imaging, ultrasound, vibro-acoustography

## Abstract

**Introduction:**

Vibro-acoustography (VA) is a recently developed imaging modality that is sensitive to the dynamic characteristics of tissue. It detects low-frequency harmonic vibrations in tissue that are induced by the radiation force of ultrasound. Here, we have investigated applications of VA for in vivo breast imaging.

**Methods:**

A recently developed combined mammography-VA system for in vivo breast imaging was tested on female volunteers, aged 25 years or older, with suspected breast lesions on their clinical examination. After mammography, a set of VA scans was acquired by the experimental device. In a masked assessment, VA images were evaluated independently by 3 reviewers who identified mass lesions and calcifications. The diagnostic accuracy of this imaging method was determined by comparing the reviewers' responses with clinical data.

**Results:**

We collected images from 57 participants: 7 were used for training and 48 for evaluation of diagnostic accuracy (images from 2 participants were excluded because of unexpected imaging artifacts). In total, 16 malignant and 32 benign lesions were examined. Specificity for diagnostic accuracy was 94% or higher for all 3 reviewers, but sensitivity varied (69% to 100%). All reviewers were able to detect 97% of masses, but sensitivity for detection of calcification was lower (≤ 72% for all reviewers).

**Conclusions:**

VA can be used to detect various breast abnormalities, including calcifications and benign and malignant masses, with relatively high specificity. VA technology may lead to a new clinical tool for breast imaging applications.

## Introduction

As a focus of intense research, breast cancer imaging technology is evolving rapidly. For many years, mammography has been the main tool used in breast imaging and is the most widely used and recommended method. The overall sensitivity of screening mammography for women ranges from 51% to 66%; women younger than 40 years have lower detectability rates, predominantly because of the greater density of breast tissue [1].

Conventional B-mode ultrasonography (US) is increasingly used as an adjunct to mammography for breast imaging; it improves sensitivity and has a considerable role in detection of cysts and solid masses [2-6]. However, it is still associated with a large number of false negative results. The sensitivity of US for detecting ductal carcinoma *in situ *is even lower than that of mammography, which limits the usefulness of US as a screening test for breast cancer [7,8].

Although magnetic resonance imaging (MRI) has higher sensitivity compared with current breast imaging methods, its lower specificity leads to unnecessary follow-up examinations and biopsies. In addition, refusal by some women to undergo MRI, the limited availability and high cost are major constraints of breast MRI [5,9,10].

To overcome the limitations of current breast imaging tools, new tools for breast imaging and evaluation must be developed. Ideally, such tools should have high sensitivity and specificity, as well as being noninvasive, capable of detecting microcalcifications, capable of imaging dense breast tissue, and available to a large patient population at a reasonable cost. Significant effort has been invested in the development and improvement of breast imaging techniques, especially those that provide palpation-like information, for example, information about tissue stiffness. The rationale for such methods is the fact that breast lesions are often stiffer than healthy tissue [11]; further, malignant lesions are stiffer than benign lesions [12,13]. Examples of imaging techniques that are sensitive to tissue stiffness include magnetic resonance elastography [14,15], US elastography [16-19], acoustic radiation force imaging [20], and supersonic shear imaging (shear wave elastography) [21].

Here, we present the applications of vibro-acoustography (VA) [22-26] in human breast imaging. Patients with various types of breast masses and calcifications were imaged, and the efficacy (diagnostic accuracy) of the method was evaluated by comparing the responses of multiple independent reviewers with clinical data.

## Materials and methods

### General principles of VA

VA measures the vibro-acoustic response of an object to a vibrating force [25,26]. This method uses US in a way that is fundamentally different from traditional US imaging. VA harnesses the radiation force of US waves, a minute force that is generated inside tissue, to remotely vibrate tissue at a low frequency. The vibrations produce a sound that can be detected by a hydrophone (a microphone designed to receive sound through water or soft tissues) and used to produce an acoustic image that represents the object's characteristics.

VA is sensitive to tissue dynamics and tissue stiffness. Generally, VA images have high resolution (1 mm or less) and are practically free of speckle noise [27]. VA image resolution is a result of the focusing effect of US, whereas the sensitivity to tissue dynamics is because tissue vibration is a function of its overall stiffness and damping. VA converts high-frequency US energy (MHz range) to a low-frequency sound (kHz range). Therefore, VA images can relay more information than traditional US by displaying tissue properties at both ends of the frequency spectrum. Preliminary work with an experimental VA system has shown the feasibility of this approach in various tissues [28-31].

### Study subjects

Female volunteers (25 years or older), who had suspected breast lesions on their clinical examination, were eligible for the study. Pregnant women were excluded. The study was conducted under a protocol approved by the Mayo Clinic Institutional Review Board. Informed consent was obtained as a part of the study protocol. Seven lesions were categorized by the Breast Imaging-Reporting and Data System (BI-RADS) as level 3, probably benign and were therefore not biopsied. These lesions were stable on short-term follow-up and did not require subsequent biopsy. The rest of the lesions underwent ultrasound-guided core needle biopsy. A 14-gauge cutting needle was utilized. Five biopsy cores were obtained in each case.

### VA system

**Figure 1:**
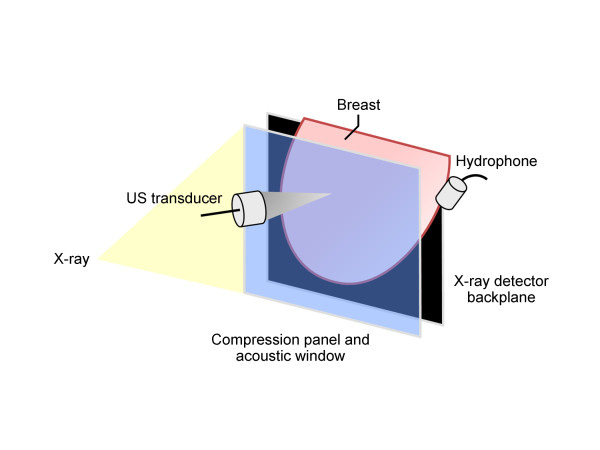
**Schematic of the experimental combined mammography and vibro-acoustic system shows the positioning of the breast**. The ultrasound (US) transducer and hydrophone are moved out of x-ray path during mammography. The compression panel has an 8 × 8-cm acoustic window covered with a latex membrane. The 5 × 5-cm imaging area is within the acoustic window.

An experimental VA system, designed for *in vivo *breast imaging, was used in this study. This system was integrated into a clinical stereotactic mammography machine (MammoTest system; Fischer Imaging, Inc, Denver, Colorado, USA) so that matching VA and mammography images (from the same view angle) could be obtained for comparison. A schematic of the system is shown in Figure 1. The system included a patient examination bed, where the patient rested in a prone position while her breast was passed through a hole in the bed. The breast was placed between the back panel, which included an x-ray detector, and a sliding panel that kept the breast slightly compressed and secured for mammography and VA scanning. The compression panel included a window covered with a thin latex membrane that was transparent to the US beam, and the US transducer was located behind the window. VA images were acquired in the cranial-caudal view at various depths from the skin. The VA image area was 5 × 5 cm, and the breast typically was scanned in 0.2-mm steps in either direction. Image resolution, which is determined by the width of the US beam, was about 0.7 mm [23]. The hydrophone was placed on the side of the breast to receive the acoustic emission generated by the radiation force of US.

### Reference image

We (the study authors) reviewed all data accrued from each patient. The data included the mammograms obtained during the experimental protocol, other available clinical images such as clinical mammograms, US, and MRI, and clinical data, such as palpation information, from the patient record. Based on these data, the presence of the abnormality and its shape and location in the imaging window were determined. Images were reviewed to ensure that the breast stayed in the same position during mammography and VA image acquisition. The information obtained in this review was used as a reference for the blinded portion of the study.

### Blinded review

Three independent reviewers were selected to participate in a review of the VA images while masked to the patient histories and clinical data. The reviewers were two radiology residents and an assistant professor in biomedical engineering who was familiar with VA techniques. None of the reviewers had prior experience in interpretation of *in vivo *breast VA images. Because VA is a new imaging modality, it was necessary to have the reviewers undergo preliminary training to learn about VA and familiarize themselves with the general appearance of breast tissue, masses and calcification in a typical VA image. They reviewed VA images of normal tissue and benign and malignant lesions. For training purposes, the VA images, corresponding mammograms, and clinical and pathologic information from seven patients were provided to all reviewers. These patient data were excluded from the portion of the study that determined the diagnostic accuracy of VA.

For the remaining VA images, each reviewer was asked to identify mass lesions and calcifications in the image. No other data were provided to the reviewers. Reviewers indicated their confidence level in their identification by selecting one of the following attributes: detected, not detected, or inconclusive. To quantify the location, the reviewers were asked to indicate the presence or absence of possible masses or calcifications in five regions of the image: center, upper left, upper right, lower left, and lower right. Reviewers also judged the appearance of each detected lesion and noted whether it was cancer, benign, or inconclusive.

### Data analysis

Diagnostic accuracy was assessed according to the principles of the Standards for the Reporting of Diagnostic Accuracy initiative. Specifically, sensitivity and specificity for each of the three blinded reviewers was assessed separately (that is, stratified by reviewer). The 95% CIs for binomial proportions are presented using the Score method [32]. Two patients had bilateral scans, which resulted in two observations per patient. However, because of the randomization used during the blinded review, no correlation of the results was anticipated or deemed biologically plausible. As such, the nesting of observations for these patients was not considered essential and data were analyzed as independent observations. Statistical analysis was conducted using the SAS System 9.1.3 (SAS Institute, Inc., Cary, NC, USA).

### Role of the funding source

The sponsor was not involved in study design, data collection, analysis or interpretation of data, writing of the report, or the decision to submit the paper for publication.

## Results

**Figure 2:**
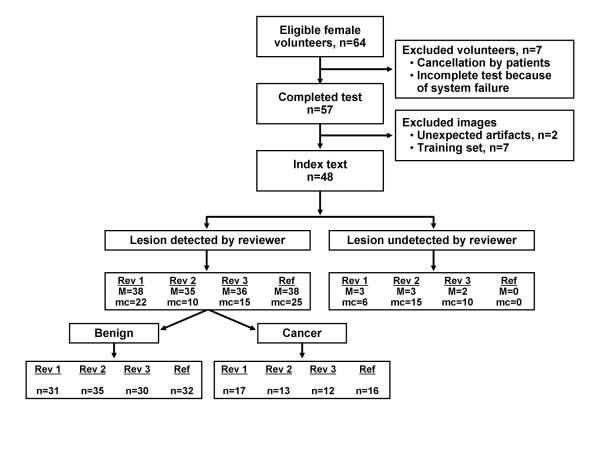
**Study flow diagram**. Reference images were read by study authors, which included a radiologist. All patient data (for example, clinical images, physical examination findings) were accessible for reference images. M, mass; mc, microcalcification; Rev, reviewer; Ref, reference.

In total, 64 women were recruited for the study. All underwent clinical mammography before participating in the study. Some patients also had breast US (*n *= 30), MRI (*n *= 10), or both (*n *= 8) before the study. VA imaging was attempted on all subjects; seven imaging attempts failed because of various technical reasons, and data from these patients were excluded from the study. The data from another seven were used for training purposes and were excluded from the blinded portion of the study. Two additional cases (both patients with fibroadenomas) were excluded from the blinded portion of the study because of unacceptable image noise levels (unknown cause). In sum, data from 48 patients were available for analysis. Figure 2 summarizes the participant classifications and exclusions.

### Diagnostic accuracy

Diagnostic accuracy was assessed by examining images and detecting masses (either benign or malignant). Mass detection was confirmed by having the reviewers indicate the location of the mass on the image. Detection of mass lesions (presence vs. absence) was uniformly high. All reviewers correctly identified 37 of the 38 images with a true mass lesion (sensitivity 97%, 95% CI 87%, 100%). All reviewers correctly located all true masses except for one fibroadenoma (for that case, two reviewers indicated the wrong location and one did not detect a mass). Specificity for mass detection ranged from 80% to 100%.

Detection of calcification was generally poor. Sensitivity for detecting calcium in the 25 positive images ranged from 40% to 72%. However, when a calcified region was identified by the reviewer, the image region also was correct in all cases. Specificity for calcification detection ranged from 83% to 87%.

In the 48 cases examined for diagnostic accuracy, 16 (33%) were classified as malignant cases (used to assess sensitivity of the VA), and the remaining 32 cases were classified as benign (used to assess specificity). Sensitivity estimates varied among reviewers (range 69% to 100%). Specificity, however, was uniformly high (≥ 94% for all reviewers) (Table 1).

**Table 1:** Diagnostic accuracy

	Sensitivity (95% CI), %			Specificity (95% CI), %		
Reference Criterion	Rev 1	Rev 2	Rev 3	Rev 1	Rev 2	Rev 3
Diagnosis						
Malignant (*n *= 16)	100 (81,100)	69 (44,86)	69 (44,86)	...	...	...
Benign (*n *= 32)	...	...	...	97 (84,99)	94 (80,98)	97 (84,99)
Mass						
Present (*n *= 38)	97 (87,100)	97 (87,100)	97 (87,100)	...	...	...
Absent (*n *= 10)	...	...	...	90 (60,98)	80 (49,94)	100 (72,100)
Calcification						
Present (*n *= 25)	72 (52,86)	40 (23,59)	60 (41,77)	...	...	...
Absent (*n *= 23)	...	...	...	83 (63,93)	87 (68,95)	87 (68,95)

CI, confidence interval; Rev, reviewer.

### Review of select cases

In this section, we present VA images of six cases and compare VA imaging results with that of other imaging modalities.

### Case 1

The patient was a woman in her 70s. A screening mammography showed scattered fibroglandular densities in each breast and multiple bilateral mass lesions (fibroadenoma).

**Figure 3:**
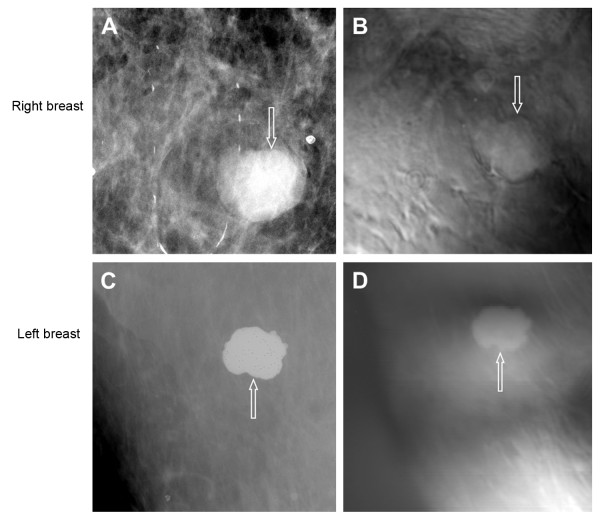
**Images from case 1**. (**A**) Prone cranial-caudal mammogram of the right breast shows a well-defined mass (arrow). (**B**) Vibro-acoustography (VA) imaging at the depth of 2.5 cm below the skin shows a well-defined mass (arrow). The slight upward shift was due to patient movement after mammography. (**C**) Prone cranial-caudal mammogram of the left breast shows a 2-cm calcified mass (arrow). (**D**) VA imaging at a depth of 2.5 cm shows the calcified mass (arrow). The slight upward shift was due to patient movement after mammography.

The prone cranial-caudal mammogram of the right breast showed a 2-cm, sharply marginated mass with coarse lobulations of soft-tissue mass (Figure 3a). The fibroadenoma region was clearly seen in the VA image, taken at a depth of 2.5 cm below the skin (Figure 3b). The VA image identified the margins well, including the gentle, coarse lobulations that are a classic finding in fibroadenoma. The mammogram additionally showed a well-circumscribed 3-mm calcification near the mass, but it was out of focus in the VA image, owing to its different depth.

The patient also had a calcified fibroadenoma in her left breast. A mammogram of the left breast showed the calcified mass (Figure 3c). The VA image clearly showed the fibroadenoma (Figure 3d). This case demonstrates that VA can identify calcified and noncalcified fibroadenoma.

### Case 2

**Figure 4:**
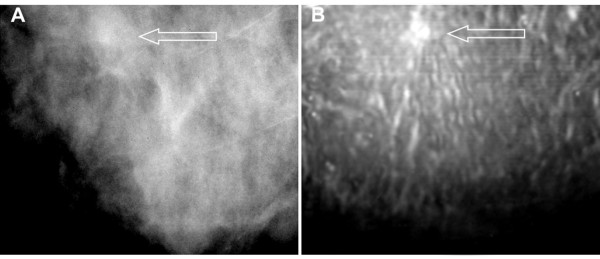
**Images from case 2**. (**A**) Mammogram shows increased soft-tissue density with undefined border (arrow). (**B**) Vibro-acoustography at a 2.0-cm depth shows an irregular mass with spiculation (arrow).

The patient was a woman in her 60s with invasive ductal carcinoma, Nottingham grade II/III, in her right breast. Screening and diagnostic mammography identified a small group of suspicious microcalcifications of varying sizes and shapes, and minimal architectural distortion and increased soft-tissue density were noted (Figure 4a). Targeted US confirmed a 5 × 7 hypoechoic lesion at the 12 o'clock position, with an irregular margin and posterior shadowing. The VA image clearly showed a small irregular mass with fine spiculation that is characteristic of this type of malignant mass (Figure 4). The characteristic spiculation was difficult to see in the mammogram. This case demonstrates that VA can identify lesions with architectural distortion (spiculation).

### Case 3

**Figure 5:**
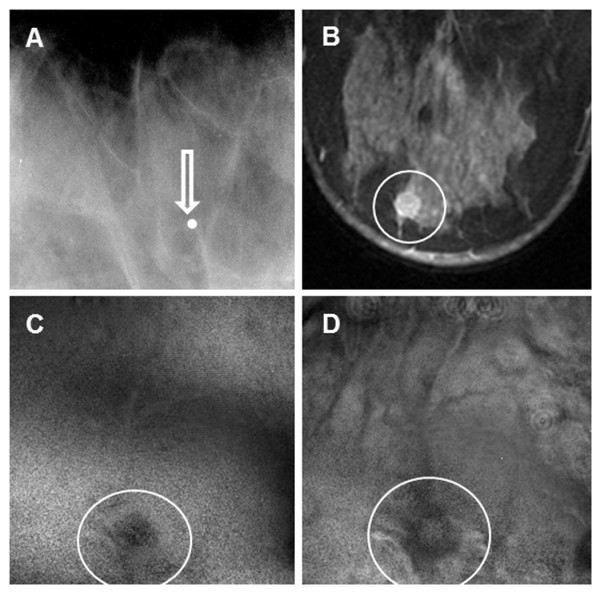
**Images from case 3**. (**A**) Mammogram shows only a marker (arrow) placed on the site of the palpable mass (no mass visible in image). (**B**) Magnetic resonance image shows the lesion (circle). (**C**-**D**) Vibro-acoustography images at a 2.5-cm depth (**C**) and 3.0-cm depth (**D**) show the lesion (circles).

The patient was a woman in her 40s with a palpable lump in the right breast. A skin marker was placed during mammography for correlation, but the mammogram showed only the marker (that is, it failed to show the lesion) because of extremely dense nodular parenchyma (Figure 5a). An MRI scan showed an abnormal, 1-cm ovoid, enhancing nodule that likely represented a fibroadenoma in the lower lateral region of the right breast, 4 cm from the nipple (Figure 5b). The nodule had an angulated margin and slightly heterogeneous, low-level internal echoes and was characterized as an indeterminate lesion. The VA image of her breast at a 2.5-cm depth (Figure 5c) and 3.0-cm depth (Figure 5d) showed the lesion; it was at the same location as determined by US and matched the marker placed during mammography. Subsequent biopsy showed a papilloma with atypia. This case demonstrates that VA can identify mammographically occult lesions in dense breast tissue.

### Case 4

**Figure 6:**
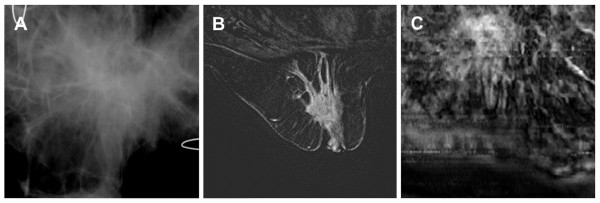
**Images from case 4**. (**A**) Mammogram shows a large distorted area with spiculation. The U-shaped wires are used to confirm image orientation. (**B**) Magnetic resonance image shows a large, irregular, and spiculated region. The main mass measured approximately 8.5 × 3.3 × 5.4 cm. (**C**) Vibro-acoustography image at a 2-cm depth shows a large spiculated mass. Note that figure parts (**A**) and (**C**) show only a 5 × 5-cm area within the breast, whereas part (**B**) shows the entire breast.

The patient was a woman in her 60s with grade I infiltrating lobular carcinoma. She had mammographically heterogeneous and dense parenchyma in both breasts. A spiculated mass with no calcification was noted in mammograms of the superior left breast (Figure 6a), with skin retraction and thickening suggestive of malignancy. Breast MRI identified a large, enhancing, irregular, spiculated mass throughout the central left breast (Figure 6b), with associated left breast shrinkage and nipple inversion. The main mass measured approximately 8.5 × 3.3 × 5.4 cm. The mass extended laterally along the pectoralis muscle. Breast VA of this patient also identified the lesion, which extended beyond the 5 × 5-cm imaging window, with remarkable spiculation suggestive of malignancy (Figure 6c). This case demonstrates that VA can identify breast cancer lesions.

### Case 5

**Figure 7:**
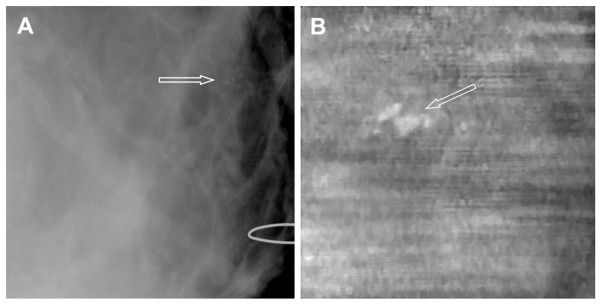
**Images from case 5**. (**A**) Mammogram shows microcalcifications that are suggestive of malignancy (arrow). (**B**) Vibro-acoustography (VA) image at 2.5-cm depth shows a cluster of calcifications (arrow). The left shift of calcification was due to patient arm movement after mammography and before VA scanning.

The patient was a woman in her 40s whose screening mammography showed heterogeneous and dense breast tissue. A diagnostic mammogram of the right breast showed pleomorphic microcalcifications that were suggestive of malignancy (Figure 7a). The VA image showed the cluster of microcalcifications with greater clarity (Figure 7b). The location of the calcification in VA was shifted leftward because the patient moved her arm after mammography, which caused the breast to shift left within the imaging window of VA scanning. The biopsy result indicated high-grade ductal carcinoma *in situ*. Microcalcifications were present in malignant ducts. This case demonstrates that VA can show clusters of malignant microcalcifications.

### Case 6

**Figure 8:**
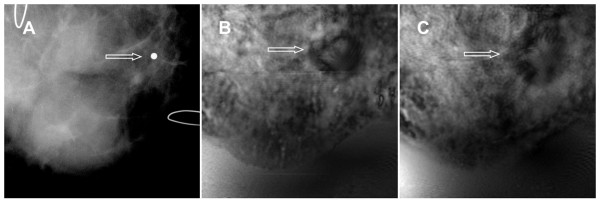
**Images from case 6**. (**A**) Mammogram shows only a marker placed in the vicinity of a palpable mass. (**B**-**C**) Vibro-acoustography images of the same breast at 2.0-cm depth (**B**) and 2.5-cm depth (**C**) show a lobulated well-defined mass (arrows).

The patient was a woman in her 40s with a palpable mass in her right breast. US examination showed a 29 × 19 × 13-mm lobulated but well-defined, mildly hypoechoic nodule with a slight increase in through-transmission. Bilateral, digital, diagnostic mammography showed heterogeneous, dense, nodular parenchyma in both breasts. The mammogram did not show the palpable mass. However, a marker was placed on the skin to identify the approximate location of the mass, as seen in the ultrasound (Figure 8a). The VA images indicated a round mass with defined border and some lobulations inside (Figure 8b and 8c). Pathologically, the mass was shown to be a fibroadenoma. This case demonstrates that VA can identify mass lesions not seen on mammograms.

## Discussion

The main goal of this study was to assess the diagnostic value of VA as a breast imaging tool. The initial results of the blinded portion of the study showed that relatively inexperienced reviewers could identify breast lesions with high specificity. However, a number of factors must be considered when interpreting these findings.

This was the first VA study of the human breast; hence, the reviewers could have only limited experience with various manifestations of breast lesions in a VA scan. After VA is used with more clinical cases and more information becomes available, skilled reviewers could be recruited for future studies. We anticipate greater experience will result in higher specificity and sensitivity values.

Because of the small sample size, we limited lesion classification to only two categories, benign and malignant. However, each class can include various subcategories with different image characteristics that, in some cases, may overlap. We speculate that a larger sample size could help improve classification and reduce errors.

The lack of speckle in VA compared with US is a considerable advantage. This allows visualization of calcifications and other small details that may otherwise be lost in a speckle-dominated US image [33].

The lesions that were identified had different contrast to the surrounding tissues. In some cases the lesions appeared brighter than the surrounding tissue (Figures 3, 4, 6, and 7), while in others the lesion appeared darker (Figures 5 and 8). Several factors contribute to the acoustic emission signal including the ultrasound reflectivity and attenuation of the tissue as well as the mechanical characteristics of the tissue at and around the focal region at the difference frequency (Δ*f*) [24-27]. Another factor that influences the response of a targeted tissue is its mechanical bonding to the surrounding tissues. The calcifications such as those in Figures 3 and 7 can be bright in the VA images because they are ultrasonically highly reflective which may increase the radiation force applied and cause the signal to increase. Soft tissue lesions may appear darker or brighter than the surrounding tissues depending on their ultrasonic and mechanical characteristics as well as their coupling to the surrounding tissue. The contrast that arises from these different types of lesions is a topic of ongoing experimental and theoretical research.

The VA system used in this study was a laboratory system sufficient for proof-of-concept examinations and exploration of the potential of VA in breast imaging. As such, this system used a mechanical drive to move a two-element transducer across the region of interest. Clinical use of VA requires a more advanced design, that is, a handheld probe with an array transducer capable of fast electronic scanning. Development and evaluation of such a clinical system is already underway [34,35]. We anticipate that this system will allow us to collect more patient data and gauge the efficacy of breast VA in clinical settings.

We acknowledge several limitations in this study. First, the reference criterion was based on the overall clinical impression and not pathologically confirmed diagnoses. Pathology results were available for 41 participants; however, ascertainment of the results was influenced by clinical impression and as such, use of the pathology results would have resulted in verification bias (biopsy was not performed in seven patients because the benignity of those lesions was certain). Second, the sample size was small and the resulting CIs were imprecise. Subsequent study is required to refine the precision of the estimates of diagnostic accuracy. Third, because the technology is new, we had considerable variation in image interpretation among reviewers in the present study. This protocol included seven cases for training purposes, but subsequent research should include more in-depth training. We anticipate that a standardized training program will decrease variation and that sensitivity will improve as future studies are conducted with larger patient groups.

Clinically, VA may be of particular use when conventional US findings are inconclusive. Further research with a larger sample size is warranted to fully assess the clinical value of this new imaging technique.

## Conclusions

*In vivo *breast imaging by VA was performed on patients with at least one breast mass. Images were evaluated by inexperienced reviewers blinded to the clinical outcomes. Sensitivity varied (depending on the target, for example, calcification, masses, or diagnosis), but the method generally demonstrated high enough diagnostic accuracy to support further exploration of the clinical value of VA in breast imaging.

## Abbreviations

BI-RADS: breast-imaging-reporting and data system; MRI: magnetic resonance imaging/image; US: ultrasonographic/ultrasonography/ultrasound; VA, vibro-acoustography.

## Competing interests

JFG, MF, and AA disclose Mayo Clinic patents on VA technology (discussed in this manuscript) as a potential financial conflict of interest. They also receive their salaries from Mayo Clinic, which is the owner of these patents. MF and JFG have received royalties from a company that has licensed the VA technology from Mayo Clinic. No organization other than Mayo Clinic College of Medicine is financing this manuscript. None of the authors currently hold stock or shares from an organization that may benefit from this manuscript.

## Authors' contributions

AA conducted the human study, and wrote most of the manuscript. DHW handled patient selection, image interpretation, and manuscript editing. MWU handled image processing, and manuscript editing. REC handled statistical design and analysis, and wrote the relevant sections. RRK acted as system technician, and operated the VA system. JFG handled technique development, and VA system design. MF handled technique development, VA system design, wrote the technical section of the paper, and supervised data acquisition and signal processing. All authors read and approved the final manuscript.
